# 3D Printing of Lotus Root‐Like Biomimetic Materials for Cell Delivery and Tissue Regeneration

**DOI:** 10.1002/advs.201700401

**Published:** 2017-10-26

**Authors:** Chun Feng, Wenjie Zhang, Cuijun Deng, Guanglong Li, Jiang Chang, Zhiyuan Zhang, Xinquan Jiang, Chengtie Wu

**Affiliations:** ^1^ State Key Laboratory of High Performance Ceramics and Superfine Microstructure Shanghai Institute of Ceramics Chinese Academy of Sciences 1295 Dingxi Road Shanghai 200050 P. R. China; ^2^ University of Chinese Academy of Sciences 19 Yuquan Road Beijing 100049 P. R. China; ^3^ Department of Prosthodontics Oral Bioengineering and Regenerative Medicine Lab Shanghai Key Laboratory of Stomatology Ninth People's Hospital affiliated to Shanghai JiaoTong University School of Medicine 639 Zhizaoju Road Shanghai 200011 P. R. China; ^4^ Oral and Maxillofacial Surgery Ninth People's Hospital affiliated to Shanghai JiaoTong University School of Medicine 639 Zhizaoju Road Shanghai 200011 P. R. China

**Keywords:** biomimetic materials, cell delivery, lotus root‐like biomaterials, tissue regeneration

## Abstract

Biomimetic materials have drawn more and more attention in recent years. Regeneration of large bone defects is still a major clinical challenge. In addition, vascularization plays an important role in the process of large bone regeneration and microchannel structure can induce endothelial cells to form rudimentary vasculature. In recent years, 3D printing scaffolds are major materials for large bone defect repair. However, these traditional 3D scaffolds have low porosity and nonchannel structure, which impede angiogenesis and osteogenesis. In this study, inspired by the microstructure of natural plant lotus root, biomimetic materials with lotus root‐like structures are successfully prepared via a modified 3D printing strategy. Compared with traditional 3D materials, these biomimetic materials can significantly improve in vitro cell attachment and proliferation as well as promote in vivo osteogenesis, indicating potential application for cell delivery and bone regeneration.

## 1 Introduction

**Figure 1:**
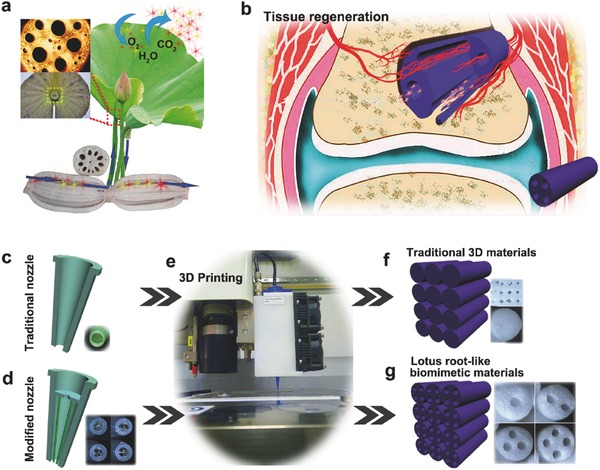
The feasible applications and fabrication of lotus root‐like biomimetic materials inspired by lotus root microstructure. a) The schemata of the functions of lotus root microstructure and the same microstructure in lotus petiole (the inset). b) The schemata of the application in tissue regeneration of lotus root‐like biomimetic materials. c) Traditional 3D printer nozzle with simple shell structure, d) the embedded structure of the modified 3D printer nozzle inspired by lotus root microstructure and e) 3D printing process. f) The traditional 3D printing scaffolds packed by solid struts. g) The lotus root‐like biomimetic materials packed by struts with different numbers of channels.

With natural evolution for millions of years, organisms have achieved multifunctional and sophisticated structures, textures or patterns spontaneously for survival, of which the unparalleled advantages have inspired the biomimetic synthesis of materials and structures in recent years. By imitating the specific hierarchical structures and synthetic process of the corresponding organisms from nanoscale to microscale, scientists have synthesized various materials with special structures and novel properties,^1^, ^2^, ^3^, ^4^, ^5^ such as ultrastrong and stiff layered composites inspired by nacre,^6^, ^7^, ^8^, ^9^, ^10^, ^11^, ^12^ high adhesion materials,^13^, ^14^ and other special biomimetic materials.^15^, ^16^, ^17^, ^18^, ^19^, ^20^, ^21^ Taking advantage of the wisdom of natural organisms, smart materials with improved properties have been prepared and used in many fields. Lotus root, a common vegetable, has unique structure of many parallel channels penetrating itself. Moreover, the parallel multichannel structure maintains in lotus petioles, which connects the lotus root and leaves to form an expedite space (**Figure** **1**a, inset). This structure significantly enlarges the contact area and keeps low flow resistance which could effectively promote air (CO_2_, O_2_) and moisture (H_2_O) exchange with external environment while reduces the weight of lotus root itself (Figure 1a). It is an ideal structure model with low density, high porosity, and low flow resistance, which is promising to be applied in bone tissue engineering (Figure 1b). The regeneration of large bone defects is still a major clinical challenge. Implantation of synthetic porous scaffolds into large bone defects is an expected approach for guiding and stimulating the formation of new bone tissue.^22^, ^23^ In addition, vascularization plays an important role in the process of new bone formation. It is known that blood vessels are initially formed as endothelial cells organizing into microtubes. Previous studies demonstrated that microfluidic system, a set of microchannels, can be used to induce endothelial cells to form rudimentary vasculature in vitro.^24^, ^25^ It has been indicated in previous studies that incorporating parts of channels through 3D scaffolds could promote oxygen/nutrient perfusion and induce tissue ingrowth along these channels.^26^, ^27^ Therefore, it is reasonable to speculate that materials with lotus root‐like structure (multichannel) possess better angiogenic and osteogenic bioactivity for the regeneration of large bone defects (Figure 1b). The 3D printing technique, which shows distinct advantages in preparing porous scaffolds with designed macropores for bone regeneration, has drawn much attention in recent years.^28^, ^29^, ^30^, ^31^, ^32^, ^33^, ^34^ However, until now, most of 3D printing scaffolds are stacked by solid struts without channel structure (Figure 1c–f). This simple structure limits the delivery of oxygen and nutrition and further the formation of new bone tissue in the center of defects.^35^, ^36^, ^37^ Meanwhile, although many 3D printing bioscaffolds with high porosity were prepared for tissue regeneration, these micropores in scaffolds cannot form channel structure, hindering the formation of rudimentary vasculature and interior new bone tissues.^38^, ^39^, ^40^, ^41^ Considering the benefits of hollow‐channel materials to vascularization and inspired by the advantages of the unique structure in lotus root, we fabricated the lotus root‐like biomimetic materials with parallel multichannels structure via a modified 3D printing strategy (Figure 1d–g; Figure S1, Supporting Information) in this study. The physicochemical properties of the lotus root‐like biomimetic materials could be effectively controlled and the lotus root‐like structure could be applied as a passageway to support cell delivery and the formation of new blood vessels and bone tissue in the inner of materials (Figure 1b). We emphatically explored the preparation and properties of the lotus root‐like biomimetic materials in tissue engineering in comparison with traditional 3D scaffolds. It is believed that the prepared lotus root‐like biomimetic materials will be ideal materials for cell delivery and tissue regeneration.

## 2 Results and Discussion

### 2.1 Fabrication and Characterization of the Lotus Root‐Like Biomimetic Materials

**Figure 2:**
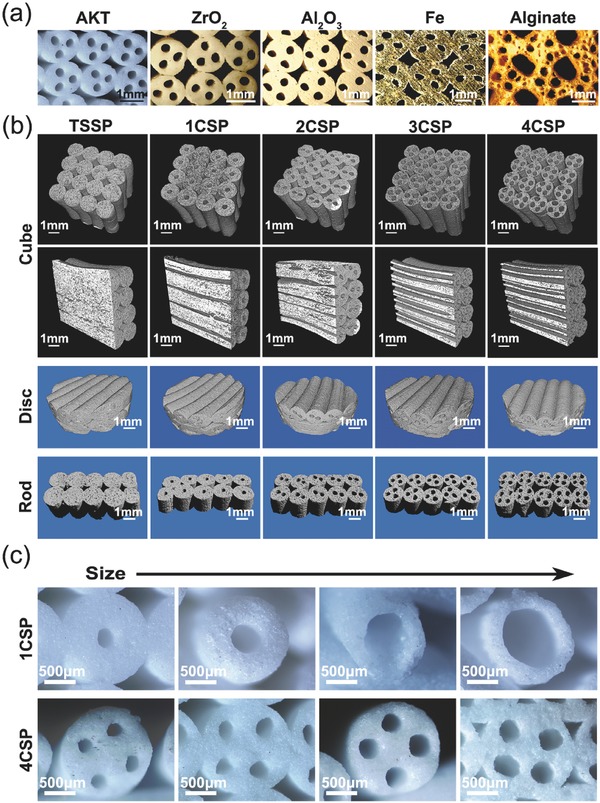
Morphology regulation and control of the lotus root‐like biomimetic materials. a) Biomimetic materials of different chemical compositions (e.g., ceramics, metal, and polymer) with lotus root‐like structure can be prepared by the modified 3D printing strategy. b) 3D micro‐CT images of biomimetic materials with different shapes (i.e., cube, disk, and rod) and different numbers of channels (e.g., 4CSP representing four channels in one strut). The shapes of biomimetic materials can be well controlled by the predesigned programs for 3D printing. c) The number and channel size can be well controlled by designing corresponding nozzle with embedded structure.

To explore the function of lotus root‐like structure in the biomimetic materials, we prepared biomimetic scaffolds with different numbers of channels: 1 channel‐struts‐packed scaffolds, 2 channel‐struts‐packed scaffolds, 3 channel‐struts‐packed scaffolds, and 4 channel‐struts‐packed scaffolds (named as 1CSP, 2CSP, 3CSP, and 4CSP scaffolds, respectively). Traditional solid struts‐packed (TSSP) scaffolds were prepared as control materials. Using this printing strategy, we directly prepared the lotus root‐like biomimetic materials at one go‐off with different raw materials including inorganic ceramic, metal, and polymer materials (**Figure** **2**a). The biomimetic materials of different chemical compositions have various morphology and properties (Figure S2, Supporting Information). We can prepare the lotus root‐like biomimetic materials with different shapes (i.e., cube, disk, and rod) to satisfy various research requirements (Figure 2b; Figure S3, Supporting Information). The number and size of channels and the dimension of struts can also be well controlled (Figure 2c; Figure S4, Supporting Information). In this study, silicate‐based bioceramic, akermanite (AKT, Ca_2_MgSi_2_O_7_) was chosen as representative biomaterial to prepare lotus root‐like biomimetic scaffolds owning to its good biocompatibility with bone marrow stromal cells according to our previous work.^28^, ^38^, ^42^

**Figure 3:**
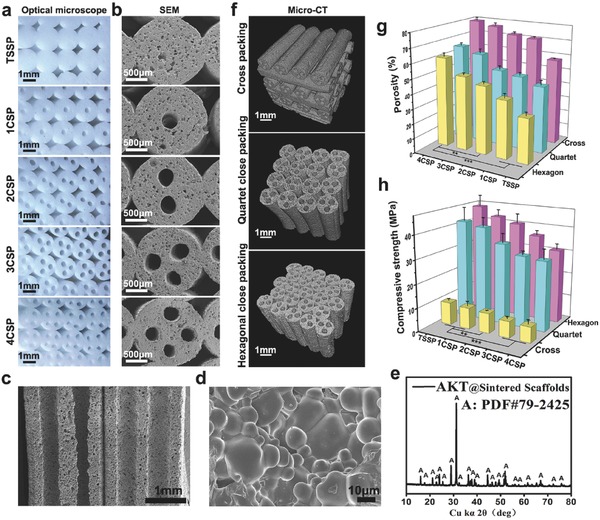
Characterizations of biomimetic silicate‐based bioceramic (Akermanite, (AKT), Ca_2_MgSi_2_O_7_) scaffolds with lotus root‐like microstructure. a) Optical microscope and b) SEM images show the lotus root‐like biomimetic structure. The materials are packed by struts (Ø1.5 mm) with different numbers of channels (Ø400–600 µm). c) SEM image for cross‐section showing that the hollow channels are completely open. d) SEM image for the inner surface microstructure of the channels. e) XRD analysis demonstrating the pure crystal phase of silicate‐based bioceramic scaffolds. f) Packing patterns (i.e., cross packing pattern, quartet close packing pattern, and hexagonal close packing pattern) have significant influence on the g) porosity and h) compressive strength of the lotus root‐like biomimetic materials. Porosity and mechanical property can be well controlled by predesign of packing pattern and number of channels. (*n* = 5, **P < 0.01, ***P < 0.001.)

As shown in the optical microscopy and scanning electron microscopy (SEM) images in **Figure** **3**a,b and Figure S5 (Supporting Information), all the scaffolds are packed by struts (Ø1.5 mm) with different numbers of channels (Ø400–600 µm). The SEM image in Figure 3c, optical microscopy images in Figure S6 (Supporting Information), and micro‐CT images of vertical section in Figure 2b all indicated that the channels in lotus root‐like structure were completely open as compared to TSSP scaffolds without round channels. The surface microstructure of the channels in the sintered biomimetic scaffolds is dense (Figure 3d). X‐ray diffraction (XRD) analysis indicates that sodium alginate and Pluronic F‐127 which were used as the binder of bioceramic ink, showed no obvious effects on the final crystal phase composition of lotus root‐like bioceramic scaffolds, which maintains pure crystal phase of Ca_2_MgSi_2_O_7_ (JCPD 79–2425) (Figure 3e). Generally, 3D scaffolds prepared by ink‐jet 3D printing technique are packed layer by layer. We adjusted the packing patterns by writing corresponding program to 3D printing system and prepared three kinds of biomimetic scaffolds with different packing patterns (i.e., cross packing pattern, quartet close packing pattern, and hexagonal close packing pattern) (Figure 3f; Figure S7, Supporting Information). With the increase of channels, the total porosity, and specific surface area significantly increased. The channel numbers can observably influence the specific surface area of the sintered scaffolds. In addition, both the channel numbers and packing patterns showed obvious impacts on porosity and compressive strength of the sintered scaffolds. The 4CSP scaffolds with cross packing pattern obtained the highest porosity of up to 80% while the porosity of TSSP scaffolds with the same packing pattern was only 58%. The biomimetic scaffolds with hexagonal close packing pattern had the best compressive strength (range of 30–46 MPa) but lower porosity than scaffolds with other two packing patterns (Figure 3g,h). The specific surface area of 4CSP scaffolds (3.86 ± 0.39 × 10^−3^ m^2^ g^−1^) was twice higher than that of TSSP scaffolds (1.40 ± 0.05 × 10^−3^ m^2^ g^−1^, Table S1, Supporting Information). Thus, much more surface area was available in the biomimetic scaffolds for cell and tissue attachment. The porosity, specific surface area, and mechanical strength of lotus root‐like biomimetic scaffolds can be well controlled by modulating the channel number or packing patterns, which is of great importance to satisfy the different mechanical requirements for human body. Therefore, we realized the controllable preparation of the biomimetic materials in both chemical compositions and physical structures.

### 2.2 In Vitro Bioactivity Analysis of the Lotus Root‐Like Biomimetic Materials

**Figure 4:**
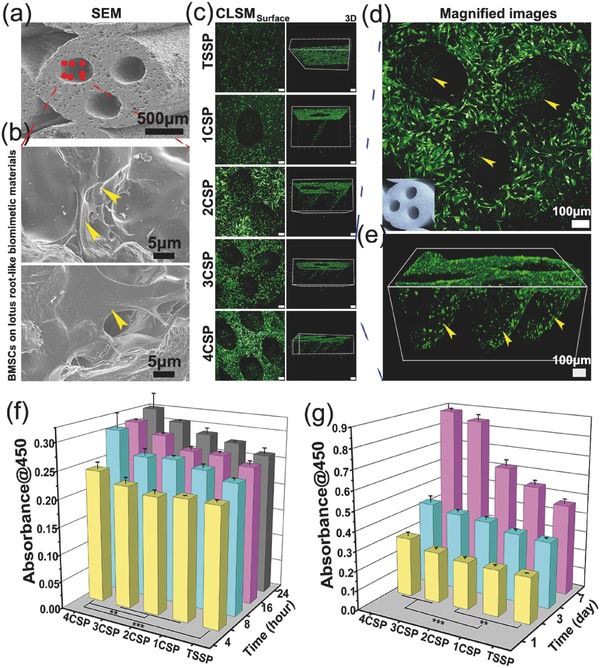
BMSCs cultured in TSSP, 1CSP, 2CSP, 3CSP, and 4CSP‐AKT bioceramic scaffolds for different time periods. a,b) SEM images of BMSCs attached in the channels of biomimetic scaffolds after culturing for 3 d. b) BMSCs adhered on the scaffolds via numerous filopodia as shown by the yellow arrows. c–e) The CLSM images for the morphology and cytoskeleton of BMSCs on the surface of struts and channels in TSSP, 1CSP, 2CSP, 3CSP, and 4CSP scaffolds after culturing for 3 d. d) Surface magnified image and e) 3D image shows that BMSCs penetrated into channels and attached on the inner walls of channels. f) The amount of adhered BMSCs after 4, 8, 16, and 24 h culturing and g) the proliferation activity of BMSCs in different scaffolds after 1, 3, and 7 d of incubation respectively, detected by the CCK‐8 assay. The initial adhered cells and their proliferation activity enhanced with the increase of the channel numbers in the biomimetic scaffolds. (*n* = 6, **P < 0.01, ***P < 0.001.)

The porous architecture and the porosity of the scaffolds play a critical role in promoting nutrient diffusion, blood vessel ingrowth, and tissue regeneration.^36^, ^43^ A potential application of the lotus root‐like biomimetic materials is bone regeneration. In this study, rabbit bone marrow stem cells (BMSCs) were seeded on the lotus root‐like biomimetic materials (1CSP, 2CSP, 3CSP, and 4CSP) with TSSP group as control. The attachment and morphology of BMSCs on the struts' surface of TSSP group and biomimetic groups were observed by SEM and confocal laser scanning microscopy (**Figure** **4**a–e; Figure S8, Supporting Information). As shown in Figure 4a,b and Figure S8a (Supporting Information), all scaffolds support BMSCs attachment and the cells closely adhere to the scaffolds by numerous filopodia after 3 d of culture. It is found that BMSCs adhere not only on the outer surface but also on the inner surface of lotus root‐like channels. As shown in Figure 4c–e and Figure S8b (Supporting Information), the cytoskeleton of BMSCs adhering on the scaffolds was stained in green with fluorescein isothiocyanate (FITC) after culturing for 3 d. The confocal laser scanning microscope (CLSM) images demonstrated that BMSCs not only attached uniformly on the surface of the scaffolds but also penetrated into the channels and attached on the walls of lotus root‐like structures (see Movies S1–S3, Supporting Information). More BMSCs were delivered in the biomimetic groups than that of TSSP group. The amount of the delivered BMSCs showed positive correlation with the number of channels in the biomimetic groups. In addition, with increasing number of hollow channels, biomimetic materials showed significant improvement on cell initial attachment at hour 8, 16, and 24 and proliferation activity at day 3 and day 7 (Figure 4f,g). The lotus root‐like structure in the biomimetic materials may be beneficial for enhancing oxygen and nutrient distribution in the inner of scaffolds. The lotus root‐like channels of the biomimetic scaffolds can be used for delivering cell and nutrition in tissue regeneration.

### 2.3 In Vivo Bioactivity Analysis of the Lotus Root‐Like Biomimetic Materials

**Figure 5:**
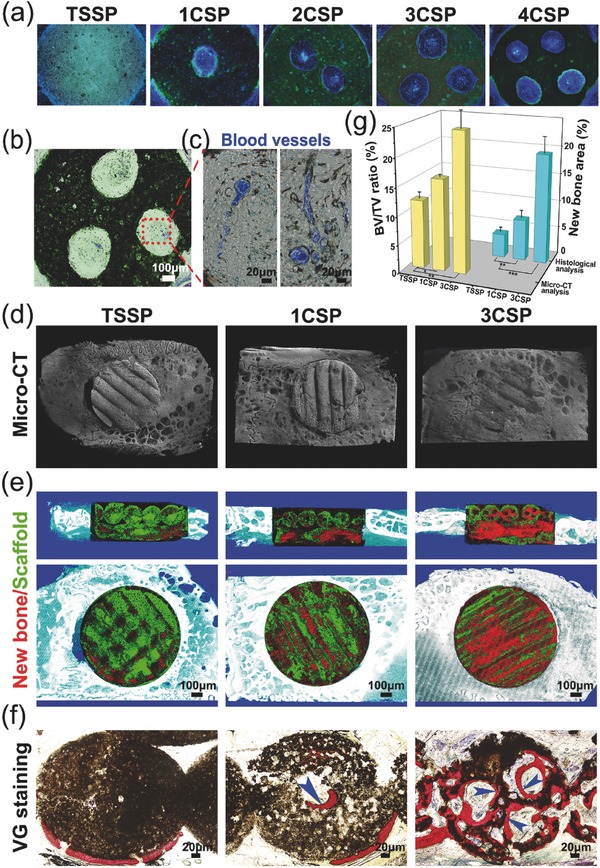
Characterizations of the lotus root‐like biomimetic scaffolds to enhance in vivo angiogenesis in rat muscle implantation and osteogenesis in rabbit calvarial defects. a) Fluorescence image of histological sections of biomimetic scaffolds stained with DAPI. b,c) The sections from microfil‐perfused samples were used to detect the new blood vessels, b) optical microscope image of 3CSP biomimetic scaffolds with blood vessels perfused by microfil, c) the magnified image of blood vessels (in blue) in the lotus root‐like structure. d) Typical 3D reconstruction micro‐CT images of the edges between materials and rabbit calvarial defects, and e) micro‐CT cross‐section images of rabbit calvarial defect regions (red for new bone tissues, green for materials). f) The undecalcified histological sections stained with Van Gieson's picrofuchsin, newly formed bone tissues (in red) can be well observed (blue arrows point to the new bone). g) Micro‐CT reconstruction analysis of the volume ratio of the newly formed bone to the defect regions (BV/TV) and histological morphometric analysis of the area of the newly formed bones in the whole defect regions at week 12. The 3CSP biomimetic materials showed significantly improvement in bone regeneration as compared to 1CSP and TSSP materials. (*n* = 6, *P < 0.05, **P < 0.01, and ***P < 0.001.)

To investigate the effect of lotus root‐like biomimetic scaffolds on the vascularization and bone regeneration, the rat muscle model and rabbit calvarial defects model were applied to evaluate both the angiogenesis and osteogenesis processes. Four weeks after implantation in the rat muscle, samples were perfused by microfil to label the blood vessels and taken for undecalcified sections, partial sections were stained with DAPI (a specific coloring agent to stain cell nucleus into blue) to detect the bioactivity of biomimetic scaffolds, other unstained sections were used to detect the newly formed blood vessels. The DAPI‐stained sections showed that the channels throughout the struts in biomimetic materials were filled with cells (**Figure** **5**a; Figure S9a, Supporting Information). The perfused microfil in the channels could be clearly detected in the histological images by different colors (blue filate tissues represent blood vessels, see Figure 5b,c). In the histological images, many blood vessels could be found in the lotus root‐like channels of the biomimetic groups while there were no blood vessels in TSSP group due to its solid struts (Figure S9b, Supporting Information). These results demonstrate that the lotus root‐like structure in the scaffolds enhances the angiogenic process at the early stage of tissue regeneration. In addition to the rat muscle implantation model, the rabbit calvarial defect model was applied to testify significantly improved osteogenesis capacity of the biomimetic materials as compared to the TSSP group. More newly formed bone tissue was observed in 3CSP group compared with 1CSP and TSSP groups after 12 weeks of implantation in the 3D micro‐CT images. The images of calvarial defect's surface showed that the bone defects in 3CSP group healed well and the peripheral bone grew tightly with the scaffolds, as compared to that of 1CSP and TSSP groups (Figure 5d). The micro‐CT images of cross‐sections showed that newly formed bone tissues had grown into the channels (Figure 5e). Besides, Van Gieson's staining results displayed that the newly formed bone tissue in the TSSP group was mainly detected in the periphery of the defects, while more newly formed bone was mainly detected in both the periphery and center of the bone defects in 1CSP and 3CSP groups, especially in the 3CSP group after implantation for 12 weeks (Figure 5f; Figure S10, Supporting Information). Moreover, according to the quantitative analysis of micro‐CT, a significantly higher BV/TV value for new bone volume was revealed for the 3CSP (24.6% ± 3.05%) group as compared with 1CSP (16.2% ± 0.503%) and TSSP (12.0% ± 1.17%) groups at week 12. Under a histomorphometric assay, the higher percentage of new bone area was observed in the 3CSP group (19.55% ± 2.88%) compared with 1CSP (7.41% ± 1.43%) and TSSP groups (4.27% ± 0.939%) at week 12 (Figure 5g). Therefore, this lotus root‐like structure can successfully induce blood vessels and new bone tissues to grow into the inner of the biomimetic materials and effectively promote the bone defect healing. The lotus root‐like biomimetic materials present better angiogenic and osteogenic stimulatory capability than traditional 3D printing scaffolds according to in vivo bioactivity analysis.

## 3 Conclusions

Inspired by the root of the natural lotus plant, we successfully prepared the biomimetic materials with lotus root‐like structure via a modified 3D printing strategy, which breaks the limitation of traditional 3D printing method. We are able to prepare the lotus root‐like biomimetic materials with different raw materials including ceramics, metal and polymer. Furthermore, their shape, packing pattern, porosity, specific surface area, mechanical property, and the lotus root‐like structure (the size and number of hollow channels and the size of struts) can be well controlled. Our results suggest that the porosity and specific surface area could be distinctly improved in the biomimetic materials. Compared to traditional 3D printing materials, the lotus root‐like biomimetic materials significantly improved in vitro BMSCs attachment and proliferation as well as in vivo osteogenesis and angiogenesis, indicating that the lotus root‐like biomimetic materials are more suitable for cell delivery and regeneration of large bone defects.

## 4 Experimental Section

*Materials*: The AKT (Ca_2_MgSi_2_O_7_), Ca, Mg, and Si‐containing bioceramic powders were synthesized by a sol–gel process using tetraethyl orthosilicate ((C_2_H_5_O)_4_Si, TEOS), magnesium dinitrate hexahydrate (Mg(NO_3_)_2_·6H_2_O), and calcium nitrate tetrahydrate (Ca(NO_3_)_2_·4H_2_O).^44^ The other ceramic and metal (ZrO_2_, Al_2_O_3_, and Fe) powders were purchased from Kunshan Chinese Technology New Materials Co., Ltd. In order to avoid blockage of the printing nozzle during the 3D printer working, the synthetic powders were ground to a particle size less than 74 µm by sieving through 200 meshes. To prepare the printable ink, 3.50 g of corresponding (AKT, ZrO_2_, Al_2_O_3_, and Fe) powders was mixed with 0.10 g of sodium alginate powder (Alfa Aesar) and 1.54 g of Pluronic F‐127 (20 wt%) (Sigma‐Aldrich) aqueous solution and then stirred until homogeneous paste was achieved.

*Design and Preparation of 3D Printer Nozzles and Printable Ink*: To design new printing nozzle for lotus root‐like biomimetic materials, different numbers of parallel needles were embedded into traditional nozzle and the number of needles embedded in the nozzle matched to the number of channels (embedded 1, 2, 3, and 4 needles for 1CSP, 2CSP, 3CSP, and 4CSP scaffolds, respectively). Simultaneously, a hole was bored through the center of every embedded nozzle to make sure the inks could be ejected out smoothly (Figure S1, Supporting Information). To prevent severe deformation or collapse of channels during the printing process, in this study, certain amounts of sodium alginate (2.0 wt%) and Pluronic F‐127 polymer solution (e.g., 20 wt%) with the under‐printed powders (ceramic/metal/polymer powders) were applied to prepare the inks with suitable rheological characteristics and mechanical stability.

*Fabrication and Characterizations of the Lotus Root‐Like Biomimetic Materials*: The 3D printing system was developed by Fraunhofer IWS (Dresden, Germany) based on the Nano‐Plotter device from GeSiM (Grosserkmannsdorf, Germany). After the scaffolds were printed, the ceramic and metal scaffolds were dried overnight at room temperature and then sintered at suitable conditions (AKT 1350 °C for 3 h, ZrO_2_, and Al_2_O_3_ 1520 °C for 3 h, Fe 1300 °C for 3 h in Ar atmosphere) to remove the sodium alginate and F‐127 phases, and the ceramic and metal particles were densified to form the final lotus root‐like biomimetic materials with certain longitudinal shrinkage and volume shrinkage. The raw polymer biomimetic materials of sodium alginate went through freeze drying process via a freeze drying machine (SRK, GMBH, Germany) to form the final scaffolds. The lotus root‐like materials still maintained the designed biomimetic structures, including macropores (outside of the struts) and open channels (inside of the struts), despite 15–25% longitudinal shrinkage and around 22–58% volume shrinkage occurring for the whole materials after high temperature sintering or freeze drying (Table S2, Supporting Information). The morphology of the sintered materials with different physicochemical properties was observed by optical microscopy (S6D, Leica, Germany). The 3D images of biomimetic materials were reconstructed by micro‐CT (SKYSCAN1172, SKYSCAN, Belgium). The macropore and microstructure of the sintered scaffolds were characterized by SEM (JSM‐6700F, JEOL, Japan). XRD (D8ADVANCE, Bruker, Germany) analysis indicated that alginate and Pluronic F‐127, as the solution of bioceramic ink, have no obvious effect on the sintered crystal phase composition of AKT bioceramics. The compressive strength of the obtained materials (10 × 10 × 15 mm) with different packing patterns and porosities were tested using a computer‐controlled universal testing machine (AG‐I, Shimadzu, Japan) at a cross‐head speed of 0.5 mm min^−1^. Five samples were tested for each kind of scaffold. The porosity was measured by Archimedes' principle, a liquid displacement method.^[45]^ In brief, the ceramic scaffolds were first dried at 100 °C overnight, weighed, and marked as *M*_1_. Then, the ceramic scaffolds were immersed in water and placed under vacuum until no bubbles appeared. The weight of the scaffolds with water‐filled pores was marked as *M*_2_. Finally, the ceramic scaffolds were immersed in water, and the buoyant weight was marked as *M*_3_. The porosity (*P*) was calculated using Equation (1)

(1) $$P=\left(M_{2}-M_{1}\right)/\left(M_{2}-M_{3}\right)\times 100\%$$

*In Vitro Bioactivity Analysis of the Lotus Root‐Like Biomimetic Materials*: BMSCs were isolated from the femurs of rabbits (one month old) and cultured in Dulbecco's modified Eagle's medium (DMEM, HyClone, China) supplemented with 10% fetal calf serum (Invitrogen) and penicillin–streptomycin (Invitrogen). For the evaluation of cell attachment, BMSCs were seeded in the lotus root‐like biomimetic scaffolds and TSSP scaffolds at an initial density of 1 × 10^4^ cells per scaffold and placed in 24‐well culture plates for culture. After incubation for 3 d, the scaffolds were rinsed with phosphate‐buffered saline (PBS) and fixed with 2.5% glutaraldehyde. The additional glutaraldehyde was removed by washing with PBS, followed by sequential dehydration in graded ethanol (30, 50, 70, 90, 95, 100, and 100 v/v%). The specimens were dried in hexamethyldisilazane for 30 min before SEM analysis. To observe the cells attached in the interior of the scaffolds, the cells were fixed with 4% paraformaldehyde solution and permeabilized in 0.1% Triton‐X‐100, and stained with FITC‐labeld Phalloidin solution (stock solution in methanol diluted in 1:100, Cytoskeleton Inc., USA) at room temperature for 45 min. Finally, after a brief PBS wash, DAPI solution (5 mg mL^−1^) was added for counterstaining cell nucleus. Confocal images were obtained on a confocal laser scanning microscope (Leica TCS SP8). CCK‐8 assay was performed to assess the initial attachment and proliferation activity of BMSCs. Briefly, BMSCs were seeded in the lotus root‐like biomimetic scaffolds and TSSP scaffolds in 24‐well culture plates and cultured for 4, 8, and 16 h and 1, 3, and 7 d. The growth medium was replaced by culture medium with CCK‐8 (1 mL, *V*_CCK‑8_:*V*_DMEM_ = 1:9) at each time point for 30 min. The absorbance was measured at λ = 450 nm in a microplate reader (Epoch microplate spectrophotometer, BioTek Instruments, USA). All the results are presented as optical density values minus the absorbance of blank wells.

*In Vivo Bioactivity Analysis of the Lotus Root‐Like Biomimetic Materials*: All experiments were performed in compliance with the relevant laws and institutional guidelines, and the Animal Care and Use Committee of Shanghai Jiaotong University approved the experiments. All animal experiments and the related experimental protocols, used in the present study, were approved by the Animal Care and Experiment Committee of the Ninth People's Hospital. To evaluate both of angiogenesis and osteogenesis processes, all total of 15 white rats and 9 New Zealand white rabbits (male, 12‐month‐old average) were taken for the model and rabbit calvarial defects model. At week 4 after the rat muscle implantation, the rats were sacrificed and perfused with microfil (Flow Tech, Carver, MA, USA). All samples were extracted and immersed in 10% buffered formaldehyde solution for 48 h and then dehydrated and embedded in polymethyl methacrylate (PMMA) for preparing the undecalcified sections. The tissue sections were made using a microtome (Leica, Nusseloch, Germany). Without staining, the sections were observed under a microscope to record the blue microfil for new blood vessels. And then, the sections were stained with DAPI (a specific coloring agent to stain cell nucleus into blue) to detect the amount of cells growing into the lotus root‐like structures. Nine New Zealand white rabbits (male, 12‐month‐old average) were taken for the rabbit calvarial defects implantation. After the general anaesthesia, an incision was made on the skull, and then the surface periosteum and Ø10 mm bone defects were completely removed from the both side of the skull, respectively, using electric drill. Three kinds of 3D printed scaffolds (disk, Ø10 × 3 mm), including 1CSP scaffold, 3CSP scaffold and TSSP scaffold, were implanted into the calvarial defects (*n* = 6). After the implantation for 12 weeks, these rabbits were sacrificed and the samples were extracted for bone regeneration evaluation. First, the samples were scanned by the Micro‐CT device and reconstructed into the three dimensional images to display the gross morphology. Afterward, the samples were dehydrated and embedded in PMMA. The undecalcified sections were made using a microtome (Leica, Nusseloch, Germany) and stained with the Van Gieson's picrofuchsin dye. On the stained sections, the percentage of the newly formed bone area in the whole defect regions was calculated.

*Statistical Analysis*: All data were displayed as the mean ± standard deviation. The statistical analysis was performed using an SAS 8.2 software. Results were analyzed for significance (*P < 0.05, **P < 0.01, and ***P < 0.001) by Student's *t‐*test (two groups) or one‐way ANOVA followed by Tukey's post hoc test for multiple comparisons.

## Conflict of Interest

The authors declare no conflict of interest.

## Supporting information

Supplementary

Supplementary

Supplementary

Supplementary

## References

[1] J. Aizenberg , P. Fratzl , Adv. Mater. 2009, 21, 387.

[2] S. E. Naleway , M. M. Porter , J. McKittrick , M. A. Meyers , Adv. Mater. 2015, 27, 5455. 26305858 10.1002/adma.201502403

[3] U. G. K. Wegst , H. Bai , E. Saiz , A. P. Tomsia , R. O. Ritchie , Nat. Mater. 2014, 14, 23. 25344782 10.1038/nmat4089

[4] G. Zan , Q. Wu , Adv. Mater. 2016, 28, 2099. 26729639 10.1002/adma.201503215

[5] B. Bhushan , Philos. Trans. R. Soc., A 2009, 367, 1445. 10.1098/rsta.2009.0011 19324719

[6] J. Zhang , W. Feng , H. Zhang , Z. Wang , H. A. Calcaterra , B. Yeom , P. A. Hu , N. A. Kotov , Nat. Commun. 2016, 7, 10701. 26907888 10.1038/ncomms10701 PMC4770083

[7] R. P. Wilkerson , B. Gludovatz , J. Watts , A. P. Tomsia , G. E. Hilmas , R. O. Ritchie , Adv. Mater. 2016, 28, 10061. 27690374 10.1002/adma.201602471

[8] H. Bai , F. Walsh , B. Gludovatz , B. Delattre , C. Huang , Y. Chen , A. P. Tomsia , R. O. Ritchie , Adv. Mater. 2016, 28, 50. 26554760 10.1002/adma.201504313

[9] G. D. Profio , S. M. Salehi , R. Caliandro , P. Guccione , G. Nico , E. Curcio , E. Fontananova , Adv. Mater. 2016, 28, 610. 26609641 10.1002/adma.201504608

[10] G. Dwivedi , K. Flynn , M. Resnick , S. Sampath , A. Gouldstone , Adv. Mater. 2015, 27, 3073. 25855576 10.1002/adma.201500303

[11] E. Munch , D. H. Alsem , E. Saiz , A. P. Tomsia , R. O. Ritchie , Science 2008, 322, 1516. 19056979 10.1126/science.1164865

[12] Q. Cheng , M. Wu , M. Li , L. Jiang , Z. Tang , Angew. Chem., Int. Ed. 2013, 52, 3750. 10.1002/anie.201210166 23401250

[13] W. Federle , W. Baumgartner , P. Drechsler , J. M. Smith , J. R. Soc., Interface 2006, 3, 689. 16971337 10.1098/rsif.2006.0135 PMC1664653

[14] L. F. Boesel , C. Greiner , E. Arzt , A. Campo , Adv. Mater. 2010, 22, 2125. 20349430 10.1002/adma.200903200

[15] S. Wang , K. Liu , X. Yao , L. Jiang , Chem. Rev. 2015, 115, 8230. 26244444 10.1021/cr400083y

[16] Q. Xu , W. Zhang , C. Dong , T. S. Sreeprasad , Z. Xia , J. R. Soc., Interface 2016, 13, 20160300. 27628170 10.1098/rsif.2016.0300 PMC5046942

[17] G. Ciasca , M. Papi , L. Businaro , G. Campi , M. Ortolani , V. Palmieri , A. Cedola , A. D. Ninno , A. Gerardino , G. Maulucci , M. D. Spirito , Bioinspiration Biomimetics 2016, 11, 011001. 26844980 10.1088/1748-3190/11/1/011001

[18] F. Xia , L. Jiang , Adv. Mater. 2008, 20, 2842.

[19] H. L. Ferrand , F. Bouville , T. P. Niebel , A. R. Studart , Nat. Mater. 2015, 14, 1172. 26390326 10.1038/nmat4419

[20] J. W. C. Dunlop , P. Fratzl , Nat. Mater. 2015, 14, 1082. 26490212 10.1038/nmat4466

[21] D. A. Muller , L. F. Kourkoutis , M. Murfitt , J. H. Song , H. Y. Hwang , J. Silcox , N. Dellby , O. L. Krivane , Science 2008, 319, 1069. 18292338 10.1126/science.1148820

[22] G. M. Calori , E. Mazza , M. Colombo , C. Ripamonti , Injury 2011, 42, 52. 10.1016/j.injury.2011.03.046 21524745

[23] Y. Liu , J. Liu , S. H. Teoh , Biotechnol. Adv. 2013, 31, 688. 23142624 10.1016/j.biotechadv.2012.10.003

[24] C. J. Bettinger , K. M. Cyr , A. Matsumoto , R. Langer , J. T. Borenstein , D. L. Kaplan , Adv. Mater. 2007, 19, 2847. 19424448 10.1002/adma.200602487 PMC2677821

[25] K. A. Heintz , M. E. Bregenzer , J. L. Mantle , K. H. Lee , J. L. West , J. H. Slater , Adv. Healthcare Mater. 2016, 17, 2153. 10.1002/adhm.201600351 PMC5014628 27239785

[26] L. S. Wray , J. R. Kovacina , B. B. Mandal , D. F. Schmidt , E. S. Gil , D. L. Kaplan , Biomaterials 2012, 33, 9214. 23036961 10.1016/j.biomaterials.2012.09.017 PMC3479404

[27] L. Wray , K. Tsioris , E. Gi , F. Omenetto , D. Kaplan , Adv. Funct. Mater. 2013, 23, 3404. 24058328 10.1002/adfm.201202926 PMC3775390

[28] C. Wu , J. Chang , S. Ni , J. Wang , J. Biomed. Mater. Res., Part A 2006, 76, 73. 10.1002/jbm.a.30496 16224776

[29] Y. Zhang , L. Xia , D. Zhai , M. Shi , Y. Luo , C. Feng , B. Fang , J. Yin , J. Chang , C. Wu , Nanoscale 2015, 7, 19207. 26525451 10.1039/c5nr05421d

[30] S. V. Murphy , A. Atala , Nat. Biotechnol. 2014, 32, 773. 25093879 10.1038/nbt.2958

[31] N. Savage , Nature 2016, 540, S56. 27926696 10.1038/540S56a

[32] A. Khademhosseini , R. Langer , Nat. Protoc. 2016, 11, 1775. 27583639 10.1038/nprot.2016.123

[33] S. J. Hollister , Nat. Mater. 2005, 4, 518. 16003400 10.1038/nmat1421

[34] J. Li , L. He , C. Zhou , Y. Zhou , Y. Bai , F. Y. Lee , J. J. Mao , MRS Bull. 2015, 40, 145.

[35] S. Bose , S. Vahabzadeh , A. Bandyopodhyay , Mater. Today 2013, 16, 496.

[36] V. Karageorgiou , D. Kaplan , Biomaterials 2005, 26, 5474. 15860204 10.1016/j.biomaterials.2005.02.002

[37] H. Mehdizadeh , S. Sumo , E. S. Bayrak , E. M. Brey , A. Cinar , Biomaterials 2013, 34, 2875. 23357368 10.1016/j.biomaterials.2012.12.047

[38] Y. Huang , X. Jin , X. Zhang , H. Sun , J. Tu , T. Tang , J. Chang , K. Dai , Biomaterials 2009, 30, 5041. 19545889 10.1016/j.biomaterials.2009.05.077

[39] A. Liu , M. Sun , X. Y. Yang , C. Y. Ma , Y. M. Liu , X. Yang , S. G. Yan , Z. R. Gou , J. Biomater. Appl. 2016, 31, 650. 27585972 10.1177/0885328216664839

[40] L. G. Galea , M. Bohner , J. Lemaitre , T. Kohler , R. Muller , Biomaterials 2008, 29, 3400. 18495242 10.1016/j.biomaterials.2008.04.041

[41] S. Liu , F. Jin , K. Lin , J. Lu , J. Sun , J. Chang , K. Dai , C. Fan , Biomed. Mater. 2013, 8, 025008. 23428666 10.1088/1748-6041/8/2/025008

[42] Q. Liu , L. Cen , S. Yin , L. Chen , G. Liu , J. Chang , L. Cui , Biomaterials 2008, 29, 4792. 18823660 10.1016/j.biomaterials.2008.08.039

[43] Q. L. Loh , C. Choong , Tissue Eng., Part B 2013, 19, 485. 10.1089/ten.teb.2012.0437 PMC3826579 23672709

[44] C. Wu , J. Chang , Mater. Lett. 2004, 58, 2415.

[45] M. Xu , D. Zhai , J. Chang , C. Wu , Acta Biomater. 2014, 10, 463. 24071000 10.1016/j.actbio.2013.09.011

