# Supplementary material for: 3D Printing of Lotus Root‐Like Biomimetic Materials for Cell Delivery and Tissue Regeneration
Source: Adv Sci (Weinh). 2017 Oct 26;4(12):1700401. doi: 10.1002/advs.201700401 (PMC5737106; doi:10.1002/advs.201700401)
Supplement: Supplementary file 1 — Supplementary [file ADVS-4-na-s001.pdf]

Copyright WILEY-VCH Verlag GmbH & Co. KGaA, 69469 Weinheim, Germany, 2017.

ADVANCED SCIENCE
Open Access

Supporting Information

for *Adv. Sci.,* DOI: 10.1002/advs.201700401

3D Printing of Lotus Root-Like Biomimetic Materials for Cell Delivery and Tissue Regeneration

Chun Feng, Wenjie Zhang, Cuijun Deng, Guanglong Li, Jiang Chang, Zhiyuan Zhang, Xinquan Jiang, and Chengtie Wu**

Copyright WILEY-VCH Verlag GmbH & Co. KGaA, 69469 Weinheim, Germany, 2016.

Supporting Information

3D Printing of Lotus Root-Like Biomimetic Materials for Cell Delivery and Tissue Regeneration

Chun Feng [1,2], Wenjie Zhang [3], Cuijun Deng [1,2], Guanglong Li [3], Jiang Chang [1], Zhiyuan Zhang [4], Xinquan Jiang [3,*] and Chengtie Wu [1,*]

C. Feng, C. Deng, Prof. J. Chang, Prof. C. Wu

1. State Key Laboratory of High Performance Ceramics and Superfine Microstructure, Shanghai Institute of Ceramics, Chinese Academy of Sciences, 1295 Dingxi Road, Shanghai 200050, P. R. China.

C. Feng, C. Deng

2. University of Chinese Academy of Sciences, 19 Yuquan Road, Beijing 100049, P. R. China.

Dr. W. Zhang, G. Li, Prof. X. Jiang

3. Department of Prosthodontics, Oral Bioengineering and Regenerative Medicine Lab, Shanghai Key Laboratory of Stomatology, Ninth People's Hospital affiliated to Shanghai JiaoTong University, School of Medicine, 639 Zhizaoju Road, Shanghai 200011, P. R. China.

Prof. Z. Zhang

4. Oral and Maxillofacial Surgery, Ninth People's Hospital affiliated to Shanghai JiaoTong University, School of Medicine, 639 Zhizaoju Road, Shanghai 200011, P. R. China.

*** Corresponding author:**

Chengtie Wu

E-mail: chengtiewu@mail.sic.ac.cn,

Tel: +86-21-52412249, Fax: +86-21-52413903;

Xinquan Jiang

E-mail: xinquanj@aliyun.com.

C. Feng and Dr. W. Zhang share the first authors.

Table S1. The specific surface area of TSSP, 1CSP, 2CSP, 3CSP and 4CSP scaffolds respectively.

**Groups**	**TSSP**	**1CSP**	**2CSP**	**3CSP**	**4CSP**
Specific surface area(×$10^{-3}$$m^2$/g)	1.40±0.05	1.87±0.13	2.43±0.26	3.08±0.18	3.86±0.39

Table S2. Longitudinal and volume shrinkage of lotus root-like biomimetic materials of different chemical compositions.

**Materials**	**Size before sintered (mm)**	**Size after sintered (mm)**	**Longitudinal shrinkage (%)**	**Volume shrinkage (%)**
AKT	11.5 ×9.2 ×3.5	10.0 ×8.0 ×3.0	13	35.2
ZrO_2	11.5 ×9.2 ×3.5	8.5 ×6.8 ×2.6	26	59.4
Al_2O_3	11.5 ×9.2 ×3.5	9.6 ×7.7 ×2.9	16	42.1
Fe	11.5 ×9.2 ×3.5	10.8 ×8.6 ×3.3	6	17.2
Alginate	11.5 ×9.2 ×3.5	10.0 ×8.1 ×3.1	12	32.2

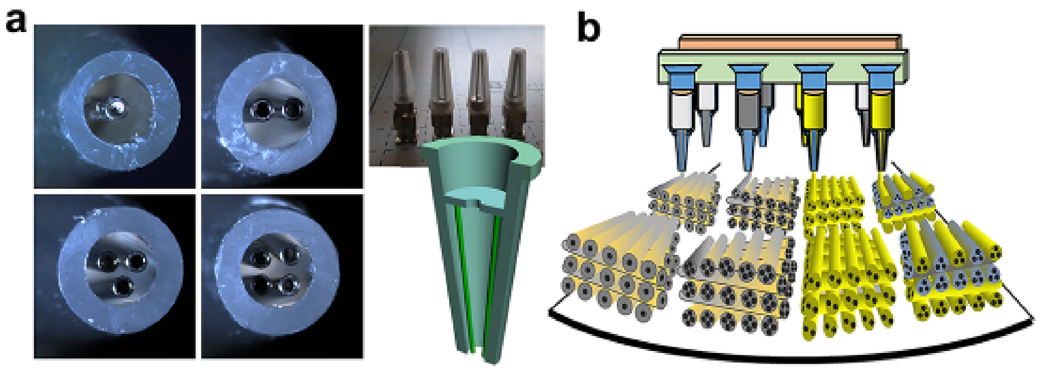

Figure S1. (a) The embedded core/shell structures of the printing nozzles for lotus root-like biomimetic materials and **(b)** the schematic diagram of 3D printing.

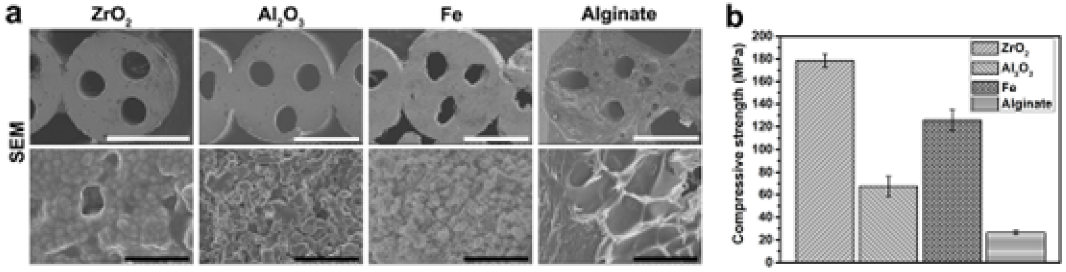

Figure S2. (a) SEM images and (b) compressive strength of lotus root-like biomimetic materials with different chemical compositions. White scale bar: 1mm, black scale bar: 5μm.

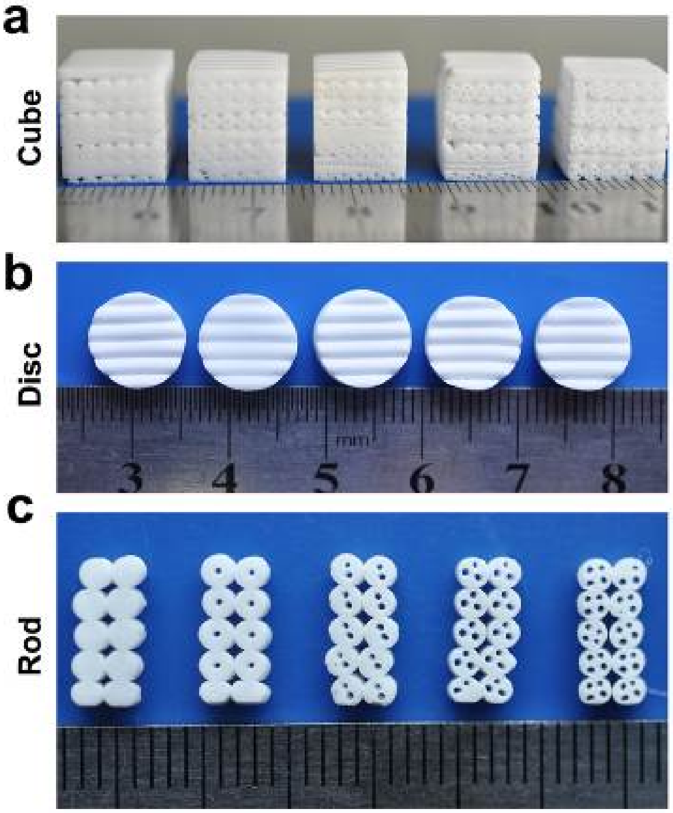

Figure S3. Lotus root-like biomimetic materials with different shapes. (a) Cube for mechanical test and adsorption test, (b) discs for regeneration of rabbit calvarial defects and (c) rods for cell attachment and proliferation and rat muscle implantation.

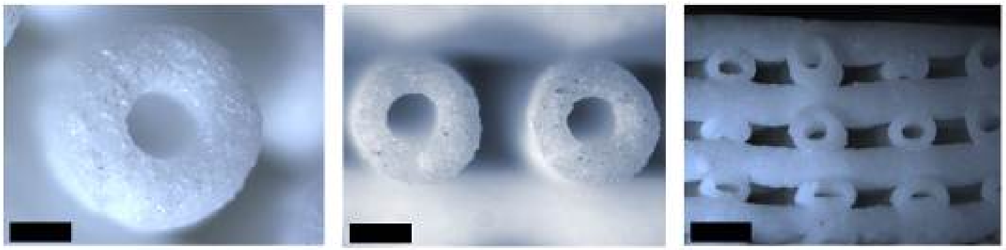

Figure S4. Lotus root-like biomimetic materials with different size of struts. Scale bar: 500μm.

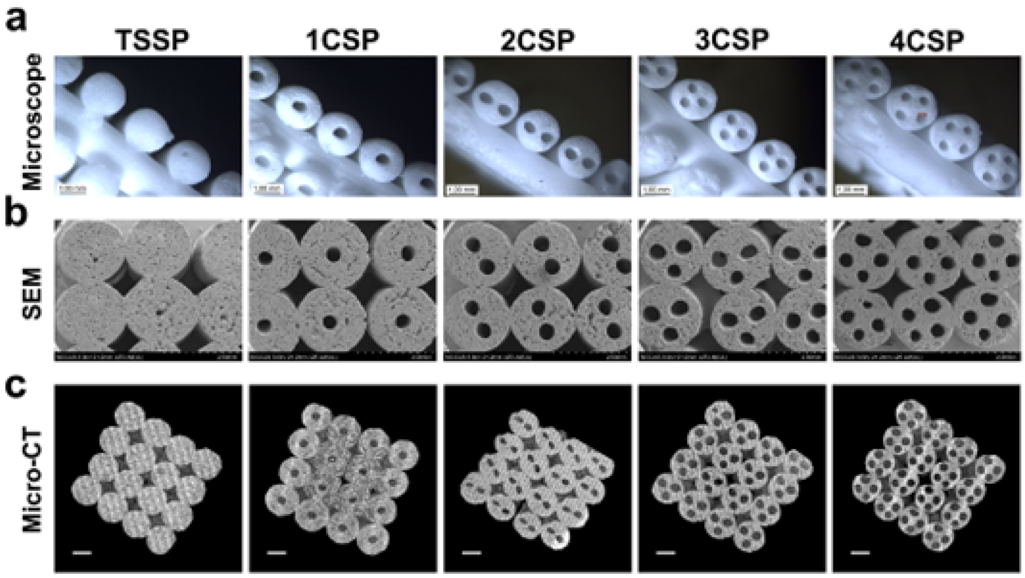

Figure S5. (a) Optical microscope, **(b)** SEM and **(c)** micro-CT images of 3D printed lotus root-like biomimetic ceramic scaffolds with same struts and channels in size. Scale bar: 1mm.

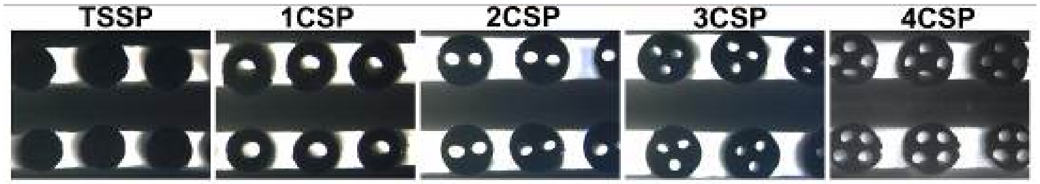

Figure S6. Optical microscope images of 3D printed lotus root-like biomimetic materials in transmission mode.

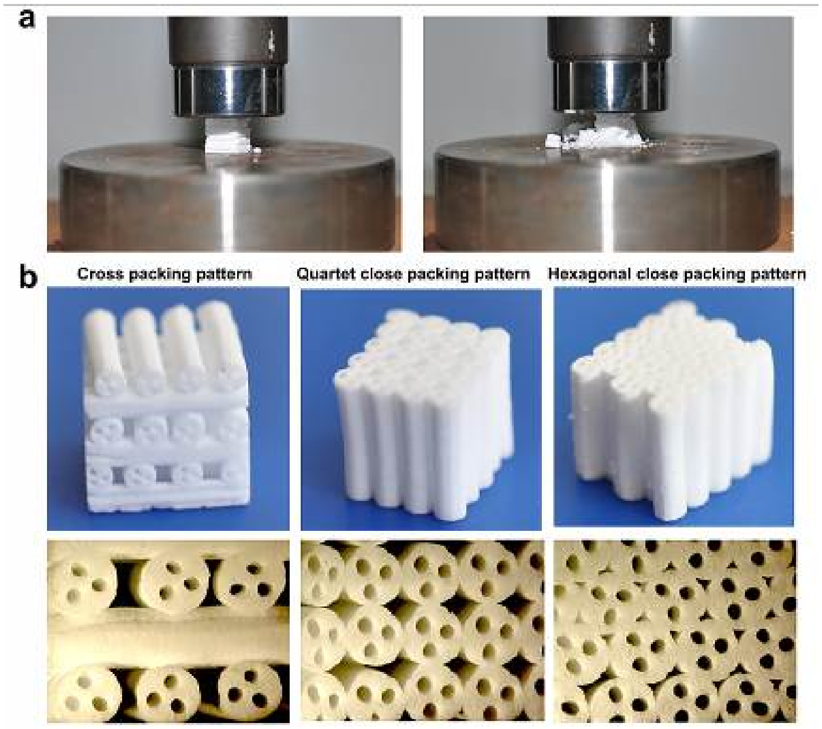

Figure S7. **(a)** Process of compressive strength test and **(b)** optical images of lotus root-like biomimetic materials with three kinds of packing patterns.

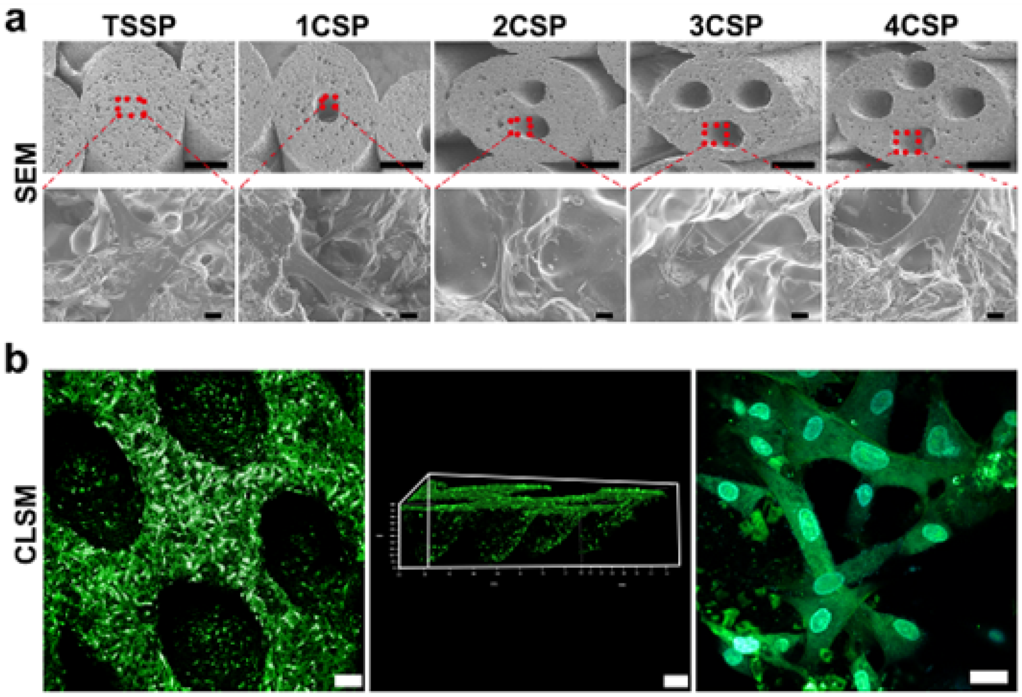

Figure S8. (a) SEM images of the morphology of BMSCs attached on the biomimetic scaffolds after culturing for 3 days and **(b)** confocal microscope images for the morphology and cytoskeleton of BMSCs on the surface and walls of 4CSP biomimetic scaffolds after culturing for 3 days. Scale bar in (a): 500μm, 5μm; scale bar in (b): 100μm, 100μm, 20μm.

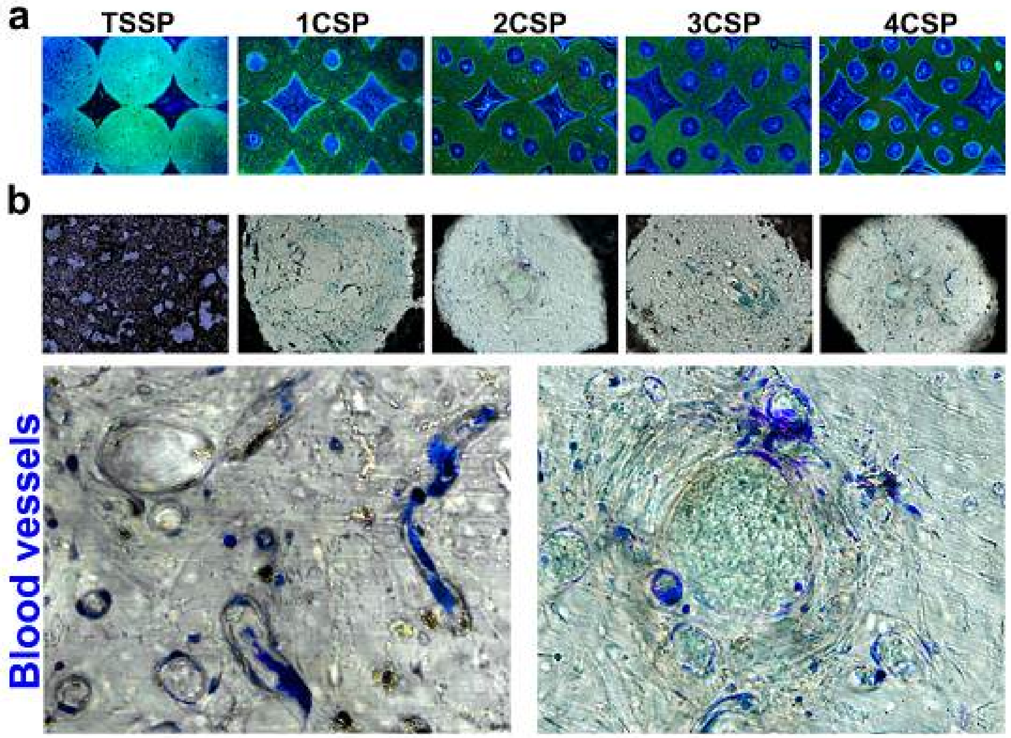

Figure S9. Histological images of the *in vivo* angiogenesis. **(a)** Histological images of biomimetic scaffolds stained with DAPI for TSSP, 1CSP, 2CSP, 3CSP and 4CSP scaffolds in rat muscle implantation after 4 weeks of implantation and **(b)** histological images of blood vessels perfused by microfil (in blue).

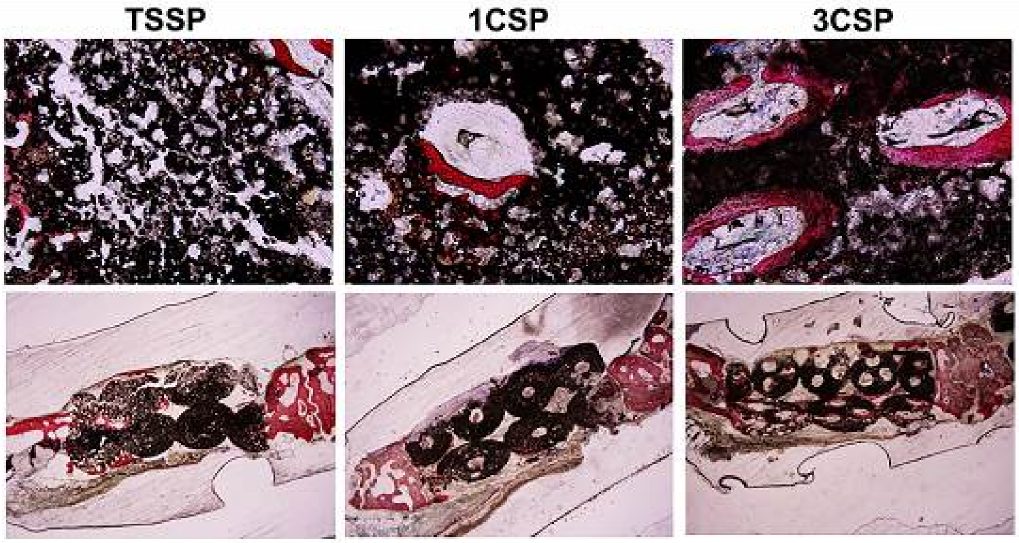

Figure S10. Histological images of newly formed bone stained with the Van Gieson's picrofuchsin dye for TSSP, 1CSP and 3CSP biomimetic scaffolds in rabbit calvarial defects

after 12 weeks of implantation.

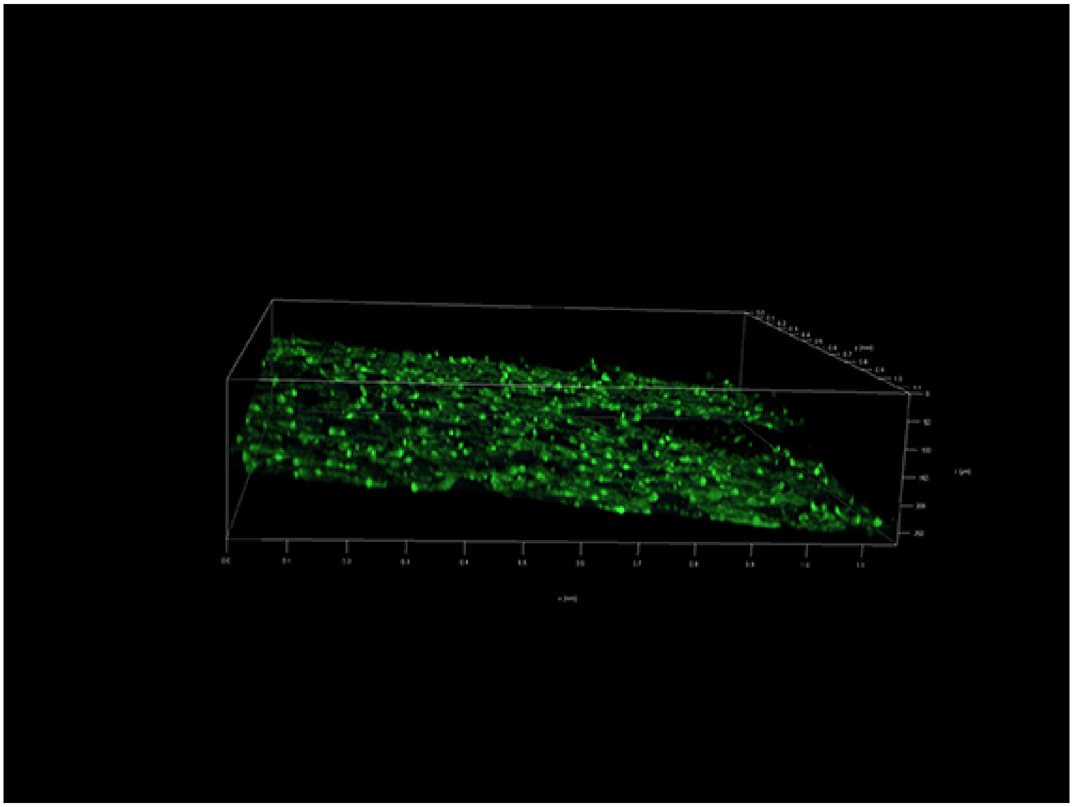

Movie S1. BMSCs on the surface of TSSP scaffolds.

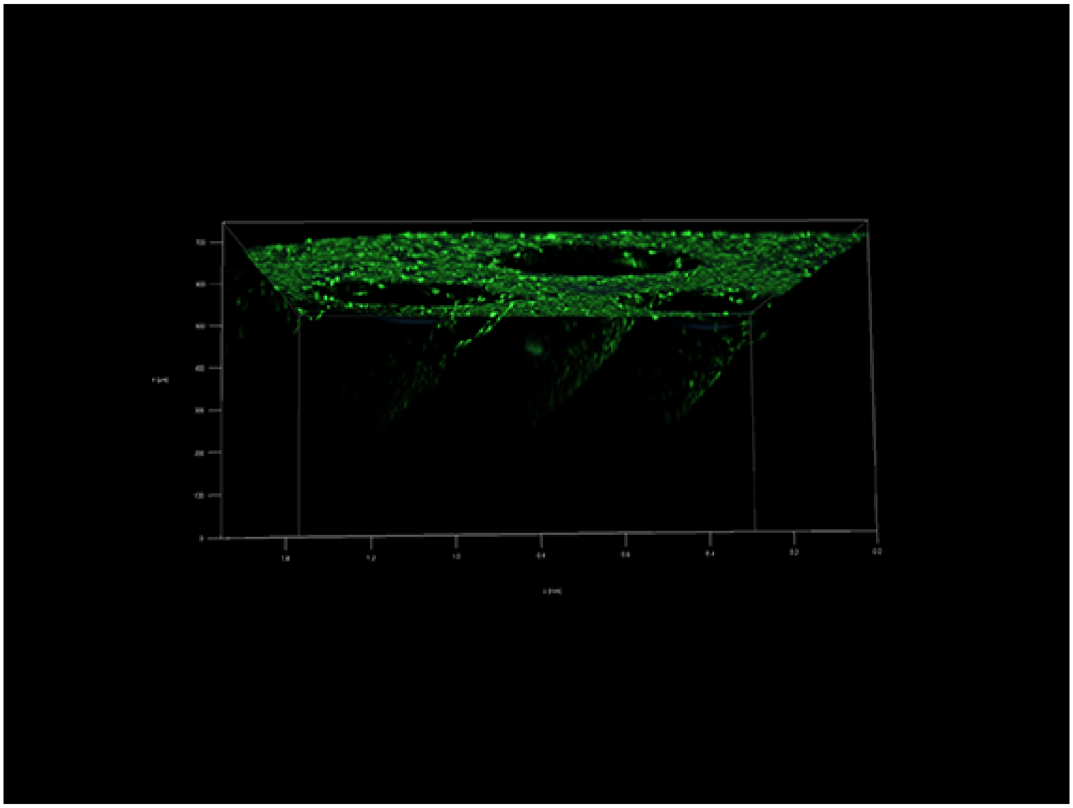

Movie S2. BMSCs on the surface and walls of 3CSP biomimetic scaffolds.

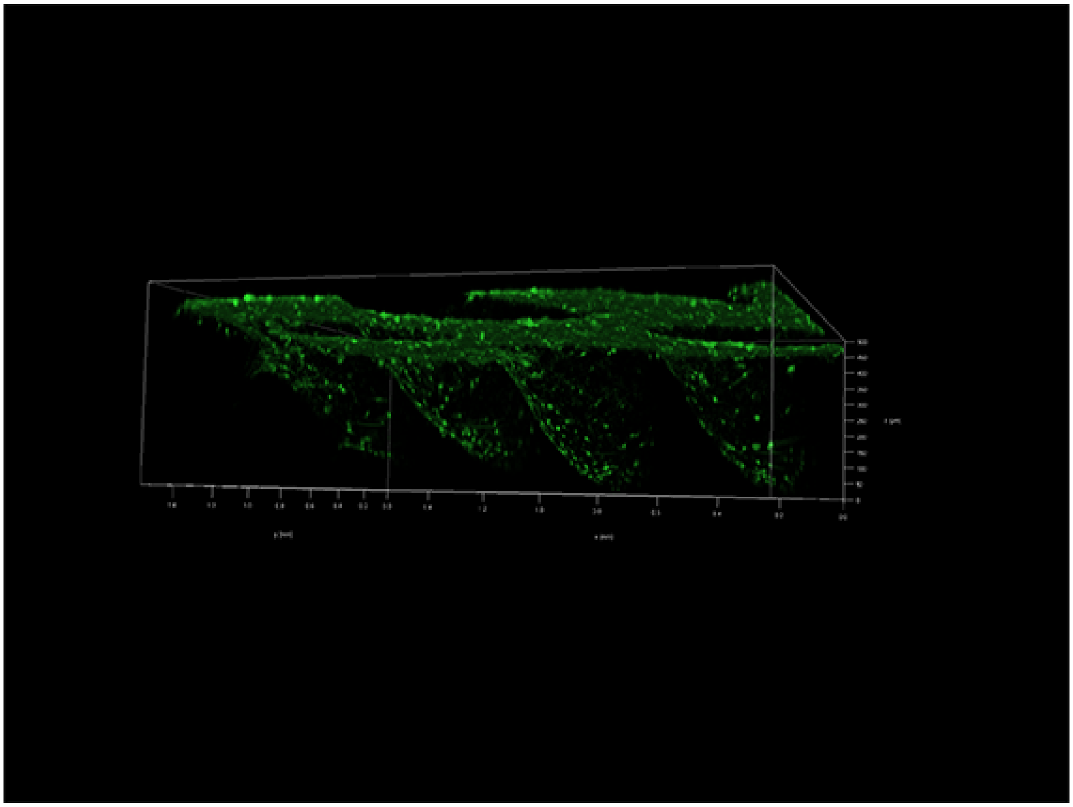

Movie S3. BMSCs on the surface and walls of 4CSP biomimetic scaffolds.
